# CoVid-19 Pandemic Trend Modeling and Analysis to Support Resilience Decision-Making

**DOI:** 10.3390/biology9070156

**Published:** 2020-07-07

**Authors:** Romney B. Duffey, Enrico Zio

**Affiliations:** 1 Independent Scholar, New York, NY 10016-5990, USA; duffeyrb@gmail.com; 2 Energy Department, Politecnico di Milano, Via La Masa 34, 20156 Milan, Italy

**Keywords:** CoVid-19, infection rates, transmission, incubation, growth, theory, predictions, recovery

## Abstract

Policy decision-making for system resilience to a hazard requires the estimation and prediction of the trends of growth and decline of the impacts of the hazard. With focus on the recent worldwide spread of CoVid-19, we take the infection rate as the relevant metric whose trend of evolution to follow for verifying the effectiveness of the countermeasures applied. By comparison with the theories of growth and recovery in coupled socio-medical systems, we find that the data for many countries show infection rate trends that are exponential in form. In particular, the recovery trajectory is universal in trend and consistent with the learning theory, which allows for predictions useful in the assistance of decision-making of emergency recovery actions. The findings are validated by extensive data and comparison to medical pandemic models.

## 1. Introduction

Today, policy decision-making for responding to the spread of CoVid-19 is based on “flattening the curve” of the accumulated number of deaths, infections and/or hospitalizations. As an example, the recent USA official guidelines [1] state that before a “comeback” phase can be initiated, in addition to some social distancing and other measures of reduction of the virus diffusion, in any given state, there must be, for a 14-day period:

- a downward trajectory of reported influenza-like illnesses (ILI).
- a downward trajectory of reported CoVid-19-like syndromic cases.

Therefore, it is fundamental to establish the significance of the downward trajectory by numerical scientific criteria that can be used by policy-makers in their region-by-region and country-by-country analyses to predict the future trend expectation and to discern significant systematic deviations from it.

For this reason, since the beginning of the spread of CoVid-19, various analytical and computational models have been developed with the aim of correlating the data of the contagion and predicting its future evolution. The early predictions were wrong, worst-case predictions neglecting the effects of countermeasures that were progressively “learned” and implemented. At worse, when assuming random infections and no countermeasures in effect, a 37% maximum infection fraction of the population was obtained by classical probability calculus [2].

In this paper, we utilized the rate of reported infection cases as a measure of the resilience response, since infections are a leading indicator of the overall trend of evolution of the pandemic in any given region. Traditionally, epidemiologists model the rate of spread using a fitted variable person-to-person transmission parameter, *R*_0_ (e.g., [3,4,5]). We provide a complete analysis for the prediction of the infection rate variation in time for the recent CoVid-19 pandemic, utilizing the precepts of the learning theory and societal system resilience. We do not consider death and hospitalization counts and rates, traditionally utilized to establish medical system loading, treatment, PPE requirements, etc., because, just as for the H1N1 virus [6] they: (i) significantly lag infections (ii) per infection case, depend highly on multiple societal factors and preexisting personal medical conditions and (iii) are subject to very variable reporting and extents, given the anticipated emergency “bed-loading” number is some fraction of the number of infections. Early predictions of massive overloading led to emergency countermeasure declarations worldwide, but the fraction of infections causing hospitalization is societally highly variable and not predetermined, depending on the medical system capability, countrywide extent, emergency treatment capacity and options and admission and discharge policies, plus any in-patient day restrictions. 

On the contrary, it is evident in the web-based data (at Johns Hopkins University (Baltimore, MD, USA), US CDC (Atlanta, GA, USA), the World Economic Forum (Cologny, Switzerland), WHO (Geneva, Switzerland), etc.) that large daily infection numbers are subject to relatively small variations, and we can, then, assume that the sample infection count can be considered typical; however, it is made or varies by testing and reporting protocols. As discussed later, in addition to statistical “spikes”, a superimposed periodicity occurs in reported infection rates, which is country and location-independent. Furthermore, using our approach, we can avoid reliance on absolute numbers and use only relative values for establishing the key trends and timescales of the infection rate evolution.

Varying national societal countermeasures or “lockdown” decrees included different “stay-at-home” orders, self-isolation or quarantine, obligatory or voluntary mask-wearing, disparate nonessential business closures, selective travel restrictions, etc. The practical purpose of analyzing and predicting the trends of growth and decline of the infection rate spread is to assist policy decision-making and verify the resilience effectiveness of such countermeasures. Here, both societal and medical system resilience are reflected and quantified in their ability and capability to reduce infection rates and to recover, not just provide, emergency treatment (Note: including pandemics, resilience has been broadly defined as: “The ability to prepare and plan for, absorb, recover from, or more successfully adapt to actual or potential adverse events” [6]). Indeed, it is found that the infection rate data, for all countries and regions for which we have data available, show a characteristic rapid growth to a peak value followed by a slower decline if countermeasures are employed/deployed effectively. To quantify these trends, we adapt the learning theory to the spread and containment of infections, as previously applied to the outcome, accident and event data for homo-technological systems. 

It is not within the scope of the present paper to establish the detailed extent of required care; rather, the objective is to provide a basis for explaining and predicting the pandemic responses and help evaluate the relative effectiveness of the countermeasures for resilience. The work presented in this paper provides a consistent theoretical and practical framework for infection rate trending and includes many useful intercomparisons, which allow:

- the verification of data reporting and trends;
- guidance to support decision-making with predicted recovery trajectories;
- the intercomparison of trends of cities, states, regions and countries;
- determination of the overall infection growth rates due to random infections followed by incubation;
- establishment of the existence or absence of consistent learning trends of recovery and the effectiveness of “lockdown” countermeasures and
- easy-to-use formulas for predictive and scoping purposes.

Other recent correlations and rate predictions have utilized the precepts of control theory [7] and, also, complex computer models of health effects [8]. We provide the clear distinctions of the present theory from those methods and direct comparisons to them.

## 2. Theoretical Framework and Data

In this section, we present the complete model for the CoVid-19 infection rate variation in time, as supported by the data from many different countries. The trend of the infection rate shows two characteristic phases:

a) initial increase up to a peak value, with a characteristic e-folding (forgetting) growth timescale due to incubation, consistent with the known case-tracking data.
b) post-peak recovery decrease from the peak, with a characteristic e-folding (learning) decline timescale dependent on societal learning and the associated countermeasures of control of the spread, consistent with the known world data.

**Figure 1:**
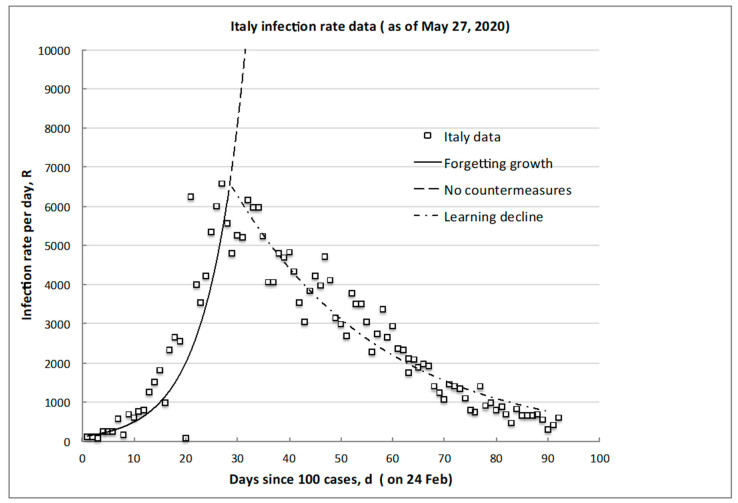
Typical infection rate trend illustrating the initial growth of the peak, *R_M_*, and desired decline to some attainable minimum, *R_m_* (CoVid-19 data for Italy).

A typical example of these phases and their trends is shown in Figure 1 using the data of Italy: the timescale is taken in days, *d*, from when 100 infections (not deaths) were first observed and reported, and the plotted infection rate, *R*, is measured in infections per day, *d* (of the infection risk exposure). It should be noted that, even after 90 days, residual or “background” infections still may occur or reemerge so the lowest infection risk achieved is not zero, which also has societal and policy implications on extending and/or maintaining countermeasures. 

We now develop the explicit equations for the growth-and-decline curves and for the peak day and other characteristic quantities of interest, based on the statistical learning theory.

### 2.1. Foundational Postulates of Learning Theory

Human decisions and actions are the dominant contributors to the many, more or less severe, accidents, crashes, system failures, medical errors and operational incidents. In the absence of a vaccine, infection events are outcomes that happen in dependence of whether humans correctly follow (learning) or not (forgetting) the established guidance, countermeasures and procedures to control the transmission and reduce the spread. Infections are, then, outcomes of events that happen because humans act correctly or incorrectly, with the postulation that humans, as they gain skills and knowledge, learn from experience in correcting or decreasing outcomes negative to them, which for the pandemic case, amounts to reducing the chance of transmission or infection. This viewpoint is consistent with the models and data of cognitive psychology of how humans behave and the brain operates in making decisions and choices [9,10] the fundamental connection being between the unconscious cognitive processes of recall and recognition and the resulting conscious actions and learned responses. The overarching postulation of the learning theory is, then, that any given human society (country, region and city) behaves and decides in a similar way when addressing pandemics, i.e., demonstrating both forgetting and learning psychological attitudes.

Another key point is that there should be no expectation of “zero biological infection risk”. The total elimination of infection opportunities likely will not occur, as learning and forgetting are continuous processes both for individuals and entire societies and, as we will see, are sometimes in a delicate balance. Therefore, statistical fluctuations, occasional subpeaks, persistent nonzero rates, recurring flare-ups and localized “hot spots” are to be expected. However, what is an acceptable or an attainable minimum infection rate, *R_m_*, remains an important societal question for policy-making.

### 2.2. Theoretical Model of Infection Rate Evolution

In the CoVid-19 diffusion, the event of infection is random, and anyone can get it by interacting with someone else. Independent of the transmission mechanism, the probability of cross-infection given contact, then, depends solely on the total number *N* of the equally infection risk-exposed individuals of the recipient population. In any observation interval considered (e.g., successive days), new infections appear as outcomes of prior or past exposures of some portion of the infection risk-exposed population. Using the principles of classical statistical mechanics [11,12,13], the rate and distribution of disease infections observed emerge from the following conventional and reasonable constraints:

- Outcomes (infections) occur and are observed randomly but are a systematic function of the risk exposure by person-to-person contact or other transmission spread processes.
- Any and all of the many distributions of infections are equally possibly occurring, with some average or overall characteristic timescale of the incubation process.
- Infections appear and are counted during some observed infection risk exposure interval (here measured in days, *d*).
- The distribution of the number of infections, *n*, which is recorded as a function of the infection risk exposure interval, is the most likely, because it is the one that has actually occurred.
- The total number of all possible infections (The magnitude of the total number of possible infections, *N*, is given by the probability of purely random unabated infection in the entire exposed population and is a maximum fraction of circa 37% (1/e), as demonstrated in [2]; *N*, and the infection risk exposure interval in days, *d*, are finite.
- The rate of infections, *R*, is proportional to the change, *dn*, in the number of infections during an incremental variation, *dd*, of the infection risk exposure interval of observation (again, taken equal to a step of 1 day, in our case).

Since the number of actual infections observed in the risk-exposure interval *d*, *n* (*d*), is (hopefully) much less than the total number of possible infections, i.e., the total population *N*, as usual for any sample *n << N*, the rate of infection is [14]

(1) $$R\left(d\right)=\frac{1}{\left(N-n\right)}\;\frac{dn}{dd}\;\approx \;\frac{1}{N}\frac{dn}{dd}$$

With the above introduced conditions, the most likely distribution of the number of infections, *n*, is, then, exponential in form [11]. Dividing this by the incremental observation interval of infection risk exposure, we obtain the observed rate, *R*(*d*), which is independent of the total number of possible infections, *N* [13] (pp. 195–197):

(2) $$R\left(d\right)=R_{m}+(R_{0}-R_{m})e^{\pm k\left(d-d_{0}\right)}$$

The positive sign describes the “forgetting” phenomenon, resulting in the rate growth due to (initially) the uncontained and/or still continuing spread until the time, *d_M_*, at which the peak rate value, *R_M_*, is reached; the negative sign gives rise to a rate decline due to (successively, day-by-day) “learning” by deploying and obeying effective countermeasures until the minimum attainable or acceptable rate value, *R_m_*, is reached. *R*_0_ is the initial rate value at the initial interval *d = d*_0_, i.e., the time of rate onset observation. The constant, *k*, is the characteristic e-folding timescale of the rate, dependent on the presence (learning) or absence (forgetting) of effective countermeasures (societal and medical, in the case of CoVid-19).

Equation (2) is the solution of

(3) $$\frac{dR}{dd}=\pm \;k\left(R-R_{m}\right)$$

which describes quantitatively the learning hypothesis that the rate of change of the rate (of reported infection cases, in this paper) is proportional to the rate itself. This formula is completely different from the rate proportionality that is arbitrarily assumed in conventional *R*_0_ epidemiological models [4].

For the initial positive exponential process of the infection rate growth, we denote positive *k = G* and consider its onset and trend in infection risk-exposure days from the initial “zero” day of spread increasing corresponding to the day, *d = d*_0_, of the first observing, say, 100 cases, which is how data is often reported. Then, Equation (2) becomes

(4) $$R\left(d\right)=R_{M}+(R_{0}-R_{M})e^{G\left(d_{0}-d\right)}$$

Similarly, for the successive negative exponential process of the infection rate decline, we use directly the symbol *k* for the exponential parameter and consider its onset and trend in infection risk-exposure days from the initial “zero” day of the spread decline, *d = d_M_*, corresponding to the day of reaching the infection rate peak, *R_M_*. Then, Equation (2) becomes

(5) $$R\left(d\right)=R_{m}+(R_{M}-R_{m})e^{-k\left(d-d_{M}\right)}$$

These growths and the decline curves (negative and positive exponentials, with parameters *G* and *k*, respectively) intersect at the peak day, *d_M_*, when the rate achieves its maximum value, *R_M_*, balancing new transmissions with prevention measures. This can sometimes generate almost a “plateau” in the actual data, depending on the absolute rate value and the actual intensity of the force of the spread and of the counterforces of the measures deployed to control it and contain it (see actual data below). From Equations (4) and (5) evaluated at their peak intersection, and considering a sufficiently long span of evolution after the onset *d >> d_0_* at which *R_M_ >> R_m_*, we obtain

(6) $$d_{M}=\left(\frac{1}{G-k}\right)\;\ln\left(\frac{R_{M}}{R_{0}-R_{m}}\right)$$

The peak rate day, *d_M_*, is, then, found to depend on the ratio of the peak rate value *R_M_* to the value of the observed rate onset, *R*_0_, and on the difference or balance between the e-folding parameters, *G* and *k*, governing the rate growth (forgetting) and decline (learning) processes, whose values are to be determined from fitting the available recorded data.

For further data intercomparisons, it is useful to adopt the nondimensional form of these trends, obtaining the so-called universal learning curve (ULC; [13]). For example, with reference to the infection rate decline phase of the learning part of the process, taking the normalized infection risk-exposure interval measure d * = (d – d_0_*)/(d_T_ − d*_0_*)*, where *d_0_ = d_M_* and *d_T_ >> d*_0_ is the total infection risk-exposure interval of the observation when learning is completed and the attainable minimum rate, *R_m_*, is reached, Equation (5) can be written [2] as

(7) $$E{\;}^{*}=\frac{R\left(d\right)-R_{m}}{R_{M}-R_{m}}=e^{-kd^{\;*}}$$

Interestingly, a value *k* ~ 3 is the rate “universally” fitting the decline trend (due to “learning”) of the failures/errors/accidents rate time series data in industrial, surgical, transportation, mining, manufacturing, chemical, maintenance, software and a multitude of other systems [13]. This same learning rate trend and constant value emerges also in cognitive psychological testing from skill acquisition tasks done by individuals, in which case, we talk of the “Universal Law of Practice” (ULP).

Similarly, for the forgetting growth part of the process, taking the normalized infection risk-exposure interval measure d * = (d_0_ *− d)/(d_M_ − d*_0_*)*, where *d_M_ >>d*_0_, Equation (4) becomes

(8) $$E^{\;*}=\frac{R\left(d\right)-R_{M}}{R_{0}-R_{M}}=e^{Gd{\;}^{*}}$$

Recently, Casella (2020) analyzed the growth and decay of infections using an empirical “control” model that is commonly used to tune the electronic system response behavior by utilizing variable feedback loops. As stated clearly by Casella [7], these arbitrary relations are not based on the first principles and “crucially depend on empirical coefficients that need to be tuned a posteriori on relevant historical data”. The limiting form of the control model ([7] Equation (10) is an exponential expression essentially identical to that which emerges from the learning hypothesis Equation (3) but has no theoretical foundation; rather, it has been fitted using (limited) infection data from Hubei, China and Lazio, Italy, obtaining a growth exponent of 3.8 days and 3.1 days, respectively. On the contrary, we have provided here the fundamentally human-societal basis for the observed trends and have shown the universal applicability of the theory on a wide range of growth and decline rate data.

### 2.3. Data Sources and Analysis 

Infection count numbers were collected daily from key official open sources on the internet covering 19th January to 30th May, 2020 and intercompared to check for consistency, specifically, unless otherwise stated:

- country-by-country data [15];
- country downloadable listings [16].

Updated analyses and predictions were made to cover the infection rates for chosen differing societal scales, localities, regions and absolute magnitudes:

○ World regions (EU, South America and USA) as a whole (interesting, because this is a globalization problem).
○ Italy as a whole and, locally, Lombardy, Lazio, Veneto and Puglia (accessed at [17]), being the largest early outbreak outside of Asia.
○ Some 14 countries with varying geographies, societies, international boundaries and countermeasures to provide comparisons (as listed in Table 1).
○ Regional Department of Health data for selected USA states with a range of infection rates and “lockdown” countermeasures (Arkansas, California, Idaho, Illinois, Iowa, Georgia, Missouri, Nebraska, New York and Wyoming).
○ Large cities or urban centers with varying population densities and countermeasures (New York, St Louis and Chicago).

The data were tabulated day-by-day for analysis and graphing purposes. The infection rates have very different values (from less than 100 per day to over 10,000 per day), depending on differences in population characteristics, countermeasures deployment timings and application effectiveness. So, it is fundamental to correctly nondimensionalize the data so that the different datasets can be intercompared and used for verifying the growth and decline trends.

The absolute value and timing of the peak rate reached also varies, as does the time to reach the minimum value, the former being typically up to 30 days and the latter 60 or more days.

We repeat that the underlying reason for considering the infection rates is the assumption that the reported testing sample or counted number of infections is some representative fraction of the actual number and is, therefore, a sample of the whole population. The number is, of course, dependent on the testing method, but the sample tested and counted is, indeed, a representative sample, whatever it is. Of course, if the testing methods are inaccurate, “underreported”, in error or change, then the uncounted/undetected fraction changes but the “missing” test or “asymptomatic” (not counted) effect is simply a systematic error continuing in the count. Furthermore, we use relative rates normalized to the initial value, so that any count fluctuations due to changing to different and/or more complex/complete testing can only appear as a small offset, as all the numbers will change correspondingly.

Differences in the reported learning process end points of the attained minimum infection rate of 50 to 100 per day are not significant with respect to the overall data variations and uncertainties relative to the peak values of the order of several 1000s per day. A previously unreported or analyzed systematic cyclical variation was observed, which we examine in Section 3.4 below.

Finally, all analyses are accompanied by statistical testing of the data fit and an estimate of the deviation bound on the normalized elapsed time, d *, to reach the maximum (*R_M_*) and minimum (*R_m_*) infection rates.

## 3. Results, Correlations and Universal Predictions

### 3.1. Growth of Transmission Trajectory

The growth to the peak of the infection rate (the solid line in the example of Figure 1) was examined for many countries/regions with discernably increasing rates. The best fit exponential was determined, the results in Table 1 being for 14 countries/regions yielding 295 data points for over 1,000,000 infections growing worldwide. The comparisons demonstrate an overall similarity of the characteristic e-folding timescale, 1/G, independent of the absolute peak infection rate value reached, which ranges over 220 < *R_M_* < 30,000 per day. The average global growth characteristic timescale value is 5.9 ± 1.4 days, with a coefficient of determination *R^2^* = 0.84 or a one standard deviation range of 4.5–7.3 days.

After the onset alarum level, no large change in the 1/G growth rate was apparent, the only common feature being all countries had similar “social distancing” pleas. The overall societal transmission and incubation timescales do not depend on the countermeasures deployed until about 30 days or more have elapsed and continuing individual infection exposure opportunities have been reduced. For example, in Italy, “stay-at-home” was decreed at day 14 (in correspondence of a rate of about 100 per day), but there was no instantaneous or later change in the slope (see Figure 1), with a lag of at least some two to three characteristic incubation timescales before the clear peak occurred at day 28 at a factor of nearly seven times higher in rate (6500 per day). Resilient emergency planning needs to know this lag and the possible rate increase to prepare before peaking and/or decline occurs.

**Figure 2:**
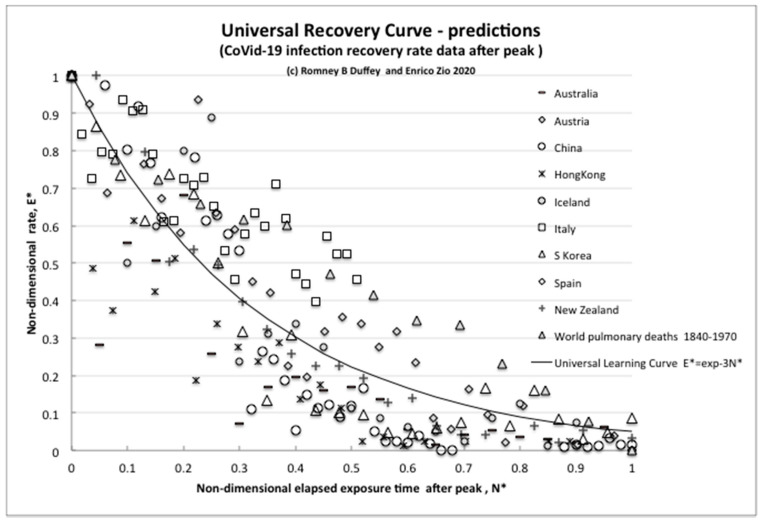
The universal recovery curve.

This characteristic timescale value for the entire society can be retained as reasonable, being comparable to the individual incubation period following infection in the USA [18]. That paper reported an average incubation time of 4.4 ± 1.7 days (these average and standard deviation were not reported in the paper, so these were calculated using the original data given in Figure 2 of the referenced paper), or between 2.7–6.1 days, from tracking cross-infections for just 15 individual cases, within the one standard deviation confidence bounds of 4.5–7.3 days determined for the global population of Table 1 and, also, the actual 1/G value for the USA of five days. In addition, a study tracking 88 individual cases in Wuhan, China [19] used a standard statistical distribution fitting for a best mean of 6.4 days, or about 5.6–7.7 days for one standard deviation, also as a range within the uncertainties. Averaging the individual and world averages gives a characteristic incubation timescale of 5.5 days.

Therefore, unsurprisingly, the world average characteristic incubation timescale for some one-million infection cases worldwide is consistent with the timescale determined locally for individuals and confirms the incubation postulation made in the theory constraints.

One can conclude that, as expected, the underlying cause of the exponentially increasing infection growth rate is, indeed, random person-to-person societal transmissions followed by incubation. Our analysis demonstrates not only that similar rapid growth is seen everywhere (Table 1) but, also, gives a timeframe for readily implementing resilient countermeasures to contain the infection spread, estimate emergency action timescales and, as we shall see, for judging their effectiveness in reducing it. 

Naturally, the peak rate, *R_M_*, and day, *d_M_*, depend also on the decline rate timescale of recovery, *k*, which is due to the countermeasures deployed as the effect of the “learning” process (Equation (6)), when the countermeasure effectiveness in growth reduction precisely balances the increases in the growth rate. Numerically, without effective recovery countermeasures, the extreme case is when *k << G*, where, for the fastest transmission, *G ~ 0.2*. So, with the nominal onset threshold of *R*_0_ = 100 per day, and no immediate effective reduction, *k ~ 0*, the plateau rate after, say, 30 days is, of course, given by *R_M_* = 100 *e* ^0.2 × 30^ ~ 40,000 per day, which is close to the observed USA maximum rate and, thus, should be expected. The ideal unattainable minimum rate is for a plateau when *k* ~ *G*, so that the instantaneous rate, *R*_0_ ~ 100, remains unaltered.

We must also examine the decline phase of the trend, and we do that by assuming a manageable minimum average rate continually attained, *R_m_*, of about 50–100 up to a few hundred per day, consistent with the available data from China, Korea, Italy and some USA states (Idaho, Wyoming and Arkansas). 

### 3.2. Decline (Recovery) Trend Predictions 

Most countries listed in Table 1 have now begun a rate decline; some have essentially completed it, and, often, a quasi-plateau of some days has been observed after reaching the peak rate value, before a steady decline starts (see the dot-dash line in Figure 1). Using such early data, even if incomplete, is important for making predictions to support effective resilience decision-making. 

As a specific example for the data from Italy in Figure 1, the growth trajectory reaching to the peak (Equation (4)) is given by $R\left(d\right)=122\;e{\;}^{0.13d\;}$, with *0 < d < d_M_,* and from Equation (6), the day of reaching the peak day is $d_{M}=\left(\frac{1}{G-k}\right)\;\ln\left(\frac{R_{M}}{R_{0}}\right)\approx \left(\frac{1}{0.14-0.025}\right)\;\ln\left(\frac{6000}{122}\right)=31$ day, which corresponds well to observation. The still-incomplete recovery trajectory of Equation (5) is given by $R\left(d\right)=17900\;e{\;}^{-0.035d\;}$, for *d* $>d_{M}$, and we may expect to reach the target nonzero minimum of circa 100 per day after about *d* = 148 days (note: at the time of original submission of this paper, the rate was circa 250 per day after *d* = 120 days).

To intercompare the recovery trajectories for different countries, we can use Equation (7) for the (normalized) universal learning curve—also called, in this case, the universal recovery curve (URC;) [2]. The results for nine countries that have demonstrably shown some form of recovery to date are reported in Figure 2, which plots E *, the nondimensional infection rate normalized to the initial peak value, versus N *, the nondimensional elapsed time of experience/knowledge or infection risk exposure after the rate has peaked (number of days after peak/day of peak).

The unexpected but intriguing result is that all countries share essentially the same exponential learning recovery trend, with slight differences. The extreme example of “perfect learning” is for the “island state” New Zealand, which follows the URC almost exactly, having the capability to completely close and control its borders against imported infections, which other regions practically cannot. Therefore, unsurprisingly, the most effective countermeasure is complete external isolation (also available for Hong Kong and Iceland), but simple internal countermeasures also work to effectively reduce the rates (as in Italy, Spain and Austria).

For further direct comparisons, and to show that this trend of behavior is not unusual, we plotted also the reduction curve of the world pulmonary disease death rate per day for 1870–1970 [20]. We can simply think of this overall world data over the years, and its reduction trend, as resulting from many pandemics and multiple outbreaks of influenzas and differing viral strains that have been more and more successfully treated as we have learned to better control/reduce infections and improved the recovery effects, thus steadily reducing the rate. Despite the huge differences in timescales, the “recovery rate curve” is still the exponential universal learning curve of Equation (7) with k ~ 3.

We can claim that this common trend decline of the URC due to learning is direct evidence of learning about risk reduction also in this case of the pandemic.

### 3.3. Comparison of the Theory to a Computational Model for Resilience Planning Purposes

To further confirm the URC general theoretical validity, we next compare to the latest projections for medical resources loads made by the complex computer modeling of infections and deaths in the USA [8] The numerical code used for the evaluations is widely used to predict local and regional health demands, and we plotted the rate of the predicted number of required hospital beds whose daily values were directly transcribed from the website graph (available at covid19.healthdata.org/united-states-of-america). The total time interval available considered a projection from a peak resource use on 15th April out to 1st July 2020, so to be consistent with the actual available country data, the equivalent infection rate per day, *R*, was calculated until attaining an assumed but realistic minimum rate, *R_m_*, of 50 per day on 10th June (55 days later).

**Figure 3:**
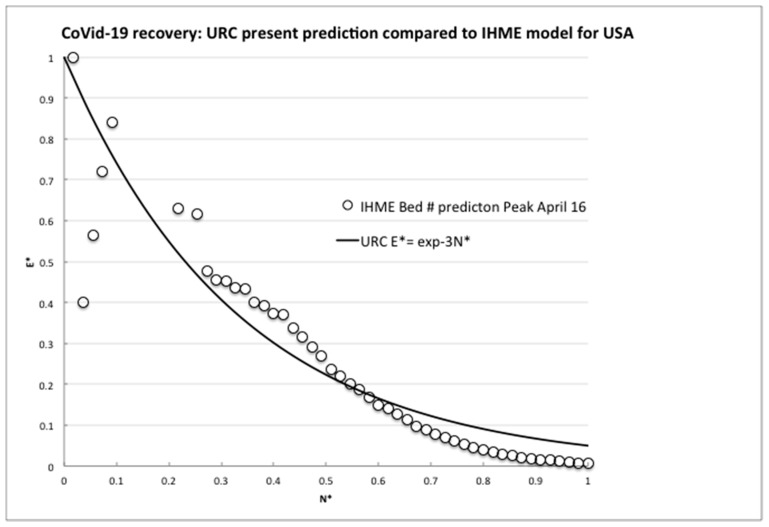
Comparison of the universal recovery curve (URC) to the model predictions of required hospital beds by IHME.

As can be seen in Figure 3, after an irregular peak, the IHME model also exhibited a steady exponential decline, although we do not know the details of the modeling assumptions. However, clearly, some effectively constant proportionality assumption was made between expected bed needs and infection numbers, because the nondimensional bed rate demand reduction closely followed the URC as demonstrated by the infection rates for many countries, not just for the USA (Figure 2). Hence, for resilience planning, the present use of the infection rate analysis is an effective leading indicator and surrogate measure for anticipated bed demands and emergency treatment capacities being proportional to the number of infections, allowing for the delay due to the above appropriate incubation timescale (five to six days) and any testing confirmations (one day).

The comparisons made here of the actually observed decreasing trends of the infection rates for nine (and still growing) independent countries and for the widely used IHME model projections with the URC shown in Figure 2 and Figure 3 above are compelling. The data fit with the predictions based on the learning theory, whose ULC we know already incidentally fits the recovery data from many events, accidents and trends. China, Italy, Spain and S. Korea have indeed learned how to control the spread of a viral pandemic, as the declining trends are showing. As to the minimum rate of, say, 100 per day (or any other number), there has to be some implicit or explicit level of socially and politically nonzero “acceptable” infection rate, as for any other disease.

### 3.4. Imperfect Learning: Plateaux, Periodicity, “Spikes” and Countermeasures Effectiveness 

This type of analysis allows countries and regions to compare the effectiveness of their countermeasures implemented to control the pandemics and the related timescales. In spite of the fact that these calculated estimates are subject to uncertainty, related to the many factors involved in the mechanisms of virus spreading and the ways that the countermeasures are implemented, they provide guidance to monitoring and predicting risks for controlling it and reducing it.

But key questions are quantifying the relative effectiveness of different countermeasures and when to declare success by “lockdown” relaxations, what low rates are attainable and the rate difference, if any, between different countermeasures. Any peak or plateau rate, *R_M_*, occurs when the countermeasure effectiveness in growth reduction precisely balances the increases in the growth rate (Equation (6)). 

We found that, for example, the USA, UK, Canada, California and Sweden also exhibited quasi-steady rate plateaus of 10 or more days, with a rate of at least 1000 per day before any steady decline, and while all included “lockdown” measures, there is no simple correlation between the plateau rate and duration. Such plateaus can be attributed to: (a) various internal regions having differing peak timing, being the summation over very large land areas and/or many cities, or (b) after having peaked, physical and societal inabilities to actually further reduce the rates. So, for resilience planning purposes, extended or flattened rate peaks can be expected, lasting many days. Countermeasures are not always equally or immediately effective or societally accepted as rapidly reducing infection rates, consistent with the five-plus-day incubation timescale and slow societal learning apparent in California and the effects of limited countermeasures in Sweden. 

**Figure 4:**
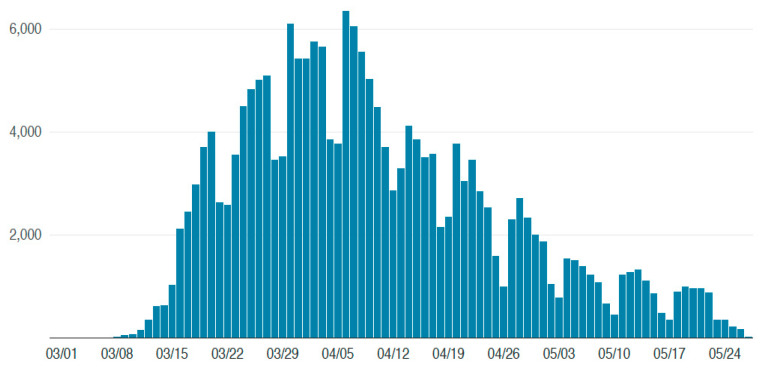
These approximately 7-day periodic peaks and troughs in the infection rate show clear minima occurring every weekend and broad peaks in the week (Source: data for New York City accessed at www1.nyc.gov/site/doh/covid/covid-19-data).

However, as opposite examples, when no such rate plateau occurred, the clear growth and, then, decline could exhibit a superimposed periodic or “saw tooth” pattern of many hundreds per day peak-to-peak (see Figure 4). The average period was determined by counting peaks and troughs, sinusoidal fitting to the residuals and by Fourier spectrum analysis. Example estimated periods were: New York City, 7 days; Italy, 6.3 days and Georgia, USA, 7.5 days, while no such periodicity was discernable in, say, the overall Russia, Brazil or Mexico data. We presume that the presence or absence of fluctuations is a result of differing societal weekly routines, testing protocols and national reporting, but also it highlights that rate variations are to be expected and short but large “spikes” of durations less than the characteristic incubation timescale are not necessarily a problem or cause for panic countermeasures.

We compared USA states with other regions that always had relatively low rates (less than 1000 per day) but with varying degrees of “lockdown” countermeasures: minimal to none in Arkansas and Sweden, strong in Denmark and Idaho, etc. There is little difference between the rates and trends for Denmark (population 5.8 M) and Arkansas (population 3 M), so the conclusion is that the simplest countermeasures suffice when there are no large potentially random infection-exposed communities or extensive travel imports. In contrast, for example, Italy (Figure 1, population 60 M) and New York (Figure 4, population 8.4 M), both with many tourists, have almost identical trends, timescales, peak rates and periodicity, so resilience planning and countermeasures were and must be much more robust for such large mobile populations.

## 4. Conclusions

In this paper, we provide a theoretical framework for the analysis and prediction of the infection rate evolution in time due to the CoVid-19 spreading. The framework is based on utilizing the learning theory, and the results obtained from the real data available support the claim that the infection rate trend decline is also direct evidence of learning about the risk reduction in this case of the pandemic. The framework and the findings of the analysis can be used to predict the expected time at which the pandemic will be under control, in terms of the minimum achievable infection rate, and to test and demonstrate the relative effectiveness of the adopted countermeasures. As such, it can be a useful tool for risk handling during the development of a pandemic and an effective measure of the desired resilience of the system. 

Significant findings include:

- the growth of the rate of infections is exponential and consistent with random transmission, and
- the decline of the rate of infections is also exponential and reflects that countermeasures have similar overall effects everywhere.

In the end, any strategy of risk governance will need to proceed rationally on the basis of:

- the acceptance of a minimum of residual risk and corresponding definition of the value of the minimum attainable/manageable infection rate,
- demonstration and verification of a steady decline in the infection rate(s), consistent with the model,
- a prediction of the time at which the minimum attainable/manageable infection rate will be reached, if not yet done,
- continuous monitoring of the situation by extended clinical testing, both systematic and random, with feedback of the test results to the predictive model,
- clear rules to identify and isolate possible local increases in the infection rate and means to manage them,
- continuous and resilient medical capacities to treat the infection cases that emerge,
- the deployment of effective, and reasonable for the situation, measures of control and containment of the virus in public and work places and
- the ethics of resilience [21]

The strategy should support gradual plans of intervention dependent on the specificity of the area and its situation of the infection process, as measured by the evolution of the infection rate. This would allow also a contained and controlled experimentation of the actions of the relaxation of containment measures before its extension to large regions and their populations.

## Figures and Tables

**Table 1:** Characteristic growth rate.

Country/State	Growth Exponent Per Day, G	Coefficient of Determination, R^2^	Peak Rate R_M_
China	0.26	0.86	4000
Belgium	0.18	0.93	2000
Brazil	0.13	0.86	3000
Canada	0.1	0.79	1600
Germany	0.18	0.88	6200
Italy	0.13	0.76	6000
Spain	0.19	0.88	10000
California	0.2	0.95	2200
S Korea	0.16	0.7	900
Sweden	0.1	0.81	730
Turkey	0.15	0.88	5100
UK	0.17	0.95	6000
USA	0.2	0.91	53000
EU	0.15	0.92	8700
S America	0.13	0.87	5500
*Average*	*0.17*	*0.86*	*7700*
